# Finding Colon Cancer- and Colorectal Cancer-Related Microbes Based on Microbe–Disease Association Prediction

**DOI:** 10.3389/fmicb.2021.650056

**Published:** 2021-03-16

**Authors:** Yu Chen, Hongjian Sun, Mengzhe Sun, Changguo Shi, Hongmei Sun, Xiaoli Shi, Binbin Ji, Jinpeng Cui

**Affiliations:** ^1^The Cancer Hospital of Jia Mu Si, Jiamusi, China; ^2^Oncological Surgery, The Central Hospital of Jia Mu Si, Jiamusi, China; ^3^Department of Thoracic Surgery, The Cancer Hospital of Jia Mu Si, Jiamusi, China; ^4^Medical Oncology, The Cancer Hospital of Jia Mu Si, Jiamusi, China; ^5^Geneis Beijing Co., Ltd., Beijing, China; ^6^Qingdao Geneis Institute of Big Data Mining and Precision Medicine, Qingdao, China; ^7^Department of Laboratory Medicine, Yantaishan Hospital of Yantai City, Yantai, China

**Keywords:** microbe–disease association, negative sample selection, linear neighborhood similarity, label propagation, information integration, colon cancer, colorectal carcinoma

## Abstract

Microbes are closely associated with the formation and development of diseases. The identification of the potential associations between microbes and diseases can boost the understanding of various complex diseases. Wet experiments applied to microbe–disease association (MDA) identification are costly and time-consuming. In this manuscript, we developed a novel computational model, NLLMDA, to find unobserved MDAs, especially for colon cancer and colorectal carcinoma. NLLMDA integrated negative MDA selection, linear neighborhood similarity, label propagation, information integration, and known biological data. The Gaussian association profile (GAP) similarity of microbes and GAPs similarity and symptom similarity of diseases were firstly computed. Secondly, linear neighborhood method was then applied to the above computed similarity matrices to obtain more stable performance. Thirdly, negative MDA samples were selected, and the label propagation algorithm was used to score for microbe–disease pairs. The final association probabilities can be computed based on the information integration method. NLLMDA was compared with the other five classical MDA methods and obtained the highest area under the curve (AUC) value of 0.9031 and 0.9335 on cross-validations of diseases and microbe–disease pairs. The results suggest that NLLMDA was an effective prediction method. More importantly, we found that Acidobacteriaceae may have a close link with colon cancer and *Tannerella* may densely associate with colorectal carcinoma.

## Introduction

Microbes are the most widespread microscopic organisms and affect many key biological processes including metabolic function and immune function (Qu et al., 2019; Sachdeva et al., 2019). There are many microbes in the human tissues, for example, skin (Fredricks, 2001), gut (Grenham et al., 2011), and lung (Cole, 1989). Normal microbial flora help the host health (Peng et al., 2018; Langella and Martín, 2019). Beneficial microbes, such as probiotics, synbiotics, and biotherapeutic agents, are effective therapeutic clues when normal microflora are disrupted (McFarland, 2000; Langella and Martín, 2019). However, the body easily gets sick when a microbial community is not balanced. Therefore, there are close associations between microbes and human diseases (Consortium, 2012; Peng et al., 2018).

Microorganisms have dense linkages with various diseases including infectious diseases and non-infectious diseases (Findley et al., 2013; Chen et al., 2017; Abu-Ali et al., 2018; Liu et al., 2019; Huang et al., 2020). For example, there is a close association between colorectal cancer and gut microbes (Heavey and Rowland, 2004; Belcheva et al., 2014). There was evidence that the changes in composition of the intestinal microbiota could induce human type 2 diabetes (Larsen et al., 2010). Toxins generated by microbes, such as *Streptococcus* and *Staphylococcus aureus*, could induce or even worsen inflammatory skin diseases (Belcheva et al., 2014). Thus, identifying the associations between microbes and diseases not only helps to characterize the pathogenesis of diseases but also provides new clues for the diagnosis and treatment of diseases (Peng et al., 2018). Although several validated microbe–disease associations (MDAs) have been reported in the Human MDA Database (HMDAD) dataset, there remains far from enough. Experimental methods to uncover new associations between two biological entities (for example, MDAs) are costly and time-consuming (Peng et al., 2017a, 2020b). Therefore, it is imperative to identify the possible disease-related microbes based on the computational models.

Based on the assumption that similar microbes tend to associate with similar diseases, computational methods are developed to predict MDAs. Ma et al. (2016) obtained the reported MDAs from documents and constructed the HMDAD. According to the computed microbe similarity, disease similarity, and known MDAs, various computational models are designed to find the associations between microbes and diseases. Chen et al. (2017) exploited the first MDA prediction method (KATZHMDA) based on the KATZ technique. Several MDA prediction models are then developed to discover the possible MDAs, for example, recommendation model based on neighbor information and MDA graph (NGRHMDA) (Huang et al., 2017), network consistency projection method (NCPHMDA) (Bao et al., 2017), network topological similarity method (NTSHMDA) (Luo and Long, 2018), adaptive boosting method (Peng et al., 2018), bi-direction similarity integration propagation method (Zhang et al., 2018), binary matrix completion method (BMCMDA), matrix decomposition method (Qu et al., 2019), and matrix factorization method combing credible negative MDA selection (Peng et al., 2020a). The above models obtained better performance for MDA prediction. Especially, the RNMFMDA method provided by Peng et al. (2020b) significantly improved MDA prediction through credible negative MDA selection based on positive-unlabeled learning (Peng et al., 2017b) and the matrix factorization with neighborhood regularization method. As such, RNMFMDA is one of the state-of-the-art MDA identification methods.

According to the recent report by EUROCARE, colon cancer and colorectal cancer demonstrated a minimal but significant increasing trend in the 5-year survival rate across the years by approximately 4–6%. More importantly, colon cancer (Terziæ et al., 2010; Ahmed, 2020) is the third most frequently diagnosed cancer in the United States. The disease is increasingly being certified now-a-days, even at an early or advanced stage. Colorectal cancer is now the fourth most widespread diagnosed cancer and the second most common cause of cancer death in the United States. Siegel et al. (2020) predicted that about 147,950 cases will be diagnosed with colorectal cancer and 53,200 will die from the cancer, including 17,930 individuals and 3,640 deaths in persons with age less than 50 years in 2020. Research studies suggest that colon cancer and colorectal cancer evolve in close associations with microbes (Garrett, 2019).

Therefore, in this manuscript, inspired by the neighborhood information method provided by Liu et al. (2020) and Peng et al. (2020a) and the neighbor propagation algorithm provided by Zhang et al. (2018), we developed an MDA prediction framework by integrating negative MDA selection, linear neighborhood similarity, label propagation, and information integration to find microbes associated with colon cancer and colorectal cancer. Firstly, microbe similarity matrix and disease similarity matrix were computed based on their Gaussian association profile (GAP) and symptom features. Secondly, the linear neighborhood similarity of microbes and diseases was calculated based on their neighborhood information, respectively. Thirdly, negative MDAs were selected according to the positive-unlabeled learning algorithm provided by Peng et al. (2020a). Fourthly, a label propagation method was designed to score all unknown microbe–disease pairs, and the scores were integrated based on the information integration method. Finally, NLLMDA was used to find the possible microbes related to colon cancer and colorectal cancer.

## Materials and Equipment

We downloaded MDAs from the HMDAD (Ma et al., 2016). The HMDAD contains 483 MDAs from 292 microbes and 39 diseases, and finally, 450 MDAs remain after preprocessing. Assume that the *i*^*th*^ microbe and the *j*^*th*^ disease are denoted as *m_i_* and *d_i_*, respectively. The associations between *n* microbes and *m* diseases are represented as a binary matrix *Y*_*(n = m)*_ where

(1) $$y_{ij}\;=\;\{\begin{array}{ll} 1 & if\;m_{i}\;associates\;with\;d_{i}\\ 0 & otherwise \end{array}$$

The elements with the values of 1 in *Y* are MDA data and taken as positive samples. The zero entities in *Y* are unknown microbe–disease pairs and taken as unlabeled samples. The microbe and disease similarity matrices are represented as *S*_*M*_ ∈ *ℝ*^*n*=*n*^ and *S*_*D*_ ∈ *ℝ*^*m*=*m*^, respectively.

## Methods

### Microbe GAP Similarity

Assume that the GAP *A*(*m*(*i*)) of a microbe *m_i_* can be denoted as the *i*^*th*^ row of the MDA matrix *Y*. For two microbes *m_i_* and *m_j_*, their GAP similarity can be defined as:

(2) $$S_{M}\left(m\left(i\right),m\left(j\right)\right)=\text{exp}\left(-\gamma_{m}{||A\left(m\left(i\right)\right)-A\left(m\left(j\right)\right)||}^{2}\right)$$

where $\gamma_{m}=\gamma_{m}^{\prime }/\left(\frac{1}{n}\sum_{k=1}^{n}{||A\left(m\left(k\right)\right)||}^{2}\right)$ denotes the normalized kernel bandwidth with parameter $\gamma_{m}^{\prime }$. The microbe similarity *S*_*M(n = n)*_ can be computed based on Eq. (2).

### Disease Similarity

#### Disease GAP Similarity

Assume that the GAP *A*(*d*(*i*)) of a disease *d_i_* can be denoted as the *j*^*th*^ column of the MDA matrix *Y*. For two diseases *d_i_* and *d_j_*, their GAP similarity can be defined as:

(3) $$S_{G}\left(d\left(i\right),d\left(j\right)\right)=\text{exp}\left(-\gamma_{d}{||A\left(d\left(i\right)\right)-A\left(d\left(j\right)\right)||}^{2}\right)$$

where $\gamma_{d}=\gamma_{d}^{\prime }/\left(\frac{1}{m}\sum_{k=1}^{m}{||A\left(d\left(k\right)\right)||}^{2}\right)$ denotes the normalized kernel bandwidth with parameter $\gamma {}_{d}^{\prime }$.

#### Disease Symptom Similarity

The disease symptom similarity matrix *S_s_* can be computed according to the method provided by Zhou et al. (2020).

The final disease similarity matrix *S*_*D(m = m)*_ can be defined based on the above two similarity measurements:

(4) $$S_{D}\left(d\left(i\right),d\left(j\right)\right)=S_{G}\left(d\left(i\right),d\left(j\right)\right)+\gamma S_{s}\left(d\left(i\right),d\left(j\right)\right)$$

where the parameter γ is used to measure the importance between the two similarity measurements.

### Negative MDA Selection

High-quality negative MDA samples help to improve MDA prediction performance. Peng et al. (2020b) designed a reliable negative MDA selection method based on positive-unlabeled learning and random walk with restart. The method significantly outperformed other MDA prediction methods and is one of the state-of-the-art negative sample selection methods. In this manuscript, we used the negative MDA extraction method provided by Peng et al. (2020b) to select reliable negative MDA samples.

### Linear Neighborhood Similarity

In association prediction area, Gaussian similarity is usually applied to evaluate similarity according to features of data points. However, the measurement is not robust to data points connecting different classes. Therefore, we assumed that each point can be reconstructed based on the linear combination of its neighborhoods and designed a linear neighborhood similarity measurement method to obtain more powerful similarity.

Suppose that *X_i_* represents the feature vector of the *i*^*th*^ microbe. We minimize the following objective function:

(5) $$\begin{array}{c} \theta_{i}={||X_{i}-\sum_{i_{j}:X_{i_{j}}\in N\left(X_{i}\right)}w_{ii_{j}}X_{i_{j}}||}^{2}\\ s.t.\sum_{i_{j}:X_{i_{j}}\in N\left(X_{i}\right)}w_{ii_{j}}=1,w_{ii_{j}}\geq 0 \end{array}$$

where *X*_*i_j_*_ denotes the *j*^*th*^ neighbor of *X_i_*, *N*(*X*_*i*_) represents the set of *K* nearest neighbors of *X_i_*, and *w*_*ii_j_*_ evaluates the reconstructive contribution of *X_i_* to *X*_*i_j_*_. Let *G*_*i*_*j*_*i*_*k*__ = (*X*_*i*_−*Xi*_*j*_)^*T*^(*X*_*i*_−*Xi*_*k*_) and θ_*i*_ be rewritten as:

(6) $$\begin{array}{c} \theta_{i}=\sum_{i_{j},i_{k}:X_{i_{j}},X_{i_{k}}\in N\left(X_{i}\right)}w_{ii_{j}}G_{i_{j}i_{k}}w_{ii_{k}}\\ s.t.\sum_{i_{j}:X_{i_{j}}\in N\left(X_{i}\right)}w_{ii_{j}}=1,w_{ii_{j}}=0. \end{array}$$

We then introduced *L_2_* norm of the weight *w_i_* to avoid over-fitting based on Tikhonov regularization. The final linear neighborhood similarity can be described as:

(7) $$\begin{array}{c} \theta_{i}=\sum_{i_{j},i_{k}:X_{i_{j}},X_{i_{k}}\in N\left(X_{i}\right)}w_{ii_{j}}G_{i_{j}i_{k}}w_{ii_{k}}+\alpha {||w_{i}||}^{2}=w_{i}^{T}\left(G^{i}+\alpha I\right)w_{i}\\ s.t.\sum_{i_{j}:X_{i_{j}}\in N\left(X_{i}\right)}w_{ii_{j}}=1,w_{ii_{j}}=0 \end{array}$$

where α is a weight used to balance the importance of the weight and the regularization terms.

We can solve Eqs. (5) and (7) to compute linear neighborhood weights and regularization linear neighborhood weights of $X_{i}^{\prime }$s neighbors based on standard quadratic programming. When *X*_*j*_∉*N*(*X*_*i*_), *w*_*ii*_ = 0. For each microbe or disease, the weights of its neighbors can be applied to represent their similarities. Thus, microbe (or disease) similarity can be computed by their linear neighborhood similarity and regularized by their linear neighborhood similarity.

### Label Propagation

In this study, we used a label propagation algorithm to find unobserved MDAs based on known MDAs, the computed microbe similarity and disease similarity. We first took microbes (or diseases) as nodes and the similarity weight *w*_*ij*_ as the edge from node *i* and node *j* and constructed a directed graph. The known MDAs were denoted as labels, which were propagated in the microbe graph. In each propagation, the labeled nodes were updated by integrating label information from their neighborhoods with the rate of β and keeping its initial label with the rate of 1−β.

Let $Y_{i}^{t}=\left\{y_{1i}^{t},y_{2i}^{t},y_{ni}^{t}\right\}$ represent the prediction association scores of *i*^*th*^ disease at time *t*, where *y^t^*_*ij*_ denotes the propensities of disease *d_j_* associated with microbe *m_i_*. The label propagation process can be defined as:

(8) $$Y_{i}^{t+1}=\beta WY_{i}^{t}+\left(1-\beta \right)Y_{i}^{0}$$

where $Y_{i}^{0}$ denotes the association profile of disease *d_i_*, and $Y_{i}^{t}$ will converge to:

(9) $$Y_{i}=\left(1-\beta \right){\left(I-\beta W\right)}^{-1}Y_{i}^{0}$$

where *Y_i_* is the final MDA score matrix based on disease *d_i_*, and the predicted entire MDA matrix can be written as:

(10) $$Y=\left(1-\beta \right){\left(I-\beta W\right)}^{-1}Y^{0}.$$

Similarly, we can conduct label propagation based on microbes.

### Information Integration

**FIGURE 1:**
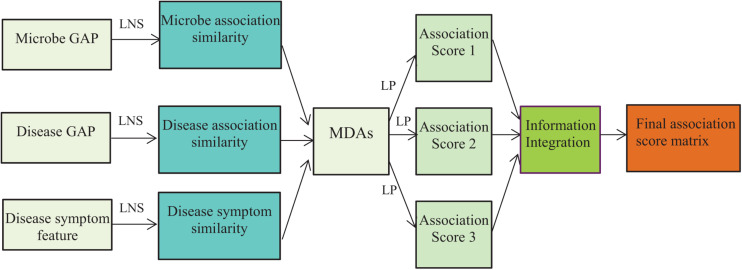
The flowchart of the NLLMDA method.

According to different features of microbes and diseases, we can compute different microbe–microbe similarities and disease–disease similarities. Different similarities produce different models and prediction results. Ensemble learning has been validated to be a powerful tool for dealing with high-dimensional and complex data. In this study, we considered diverse features of microbes and diseases and designed a linear combination technique to integrate different results. We assigned different weights to each model and integrated the predicted association scores as follows:

(11) $$\begin{array}{c} Z_{ij}=\sum_{k=1}^{S}\omega_{k}Y_{ij}^{k}\\ s.t.\sum_{k=1}^{S}\omega_{k}=1 \end{array}$$

where *S* = 3 denotes the number of different models, *Y^k^*_*ij*_ denotes the predicted association scores for microbe–disease pair (*m*_*i*_, *d*_*j*_) by the *k*^*th*^ model, ω_k_ denotes the weights of the *k*^*th*^ model, and *Z*_*ij*_ denotes the integrated association prediction score of microbe–disease pair (*m*_*i*_, *d*_*j*_). The flowchart is shown in Figure 1, where LNS and LP denote linear neighborhood similarity and label propagation.

## Results

### Experimental Settings and Evaluation Metrics

We conducted 100 trials of 5-fold cross-validation, and an average performance was calculated to decrease the prediction bias. Three different cross-validations were conducted as follows:

• 5-fold cross-validation 1 (CV1) on microbes: random rows (microbes) in MDA matrix were masked for testing.
• 5-fold cross-validation 2 (CV2) on diseases: random columns (diseases) in MDA matrix were masked for testing.
• 5-fold cross-validation 3 (CV3) on microbe–disease pairs: random entries (microbe–disease pairs) in MDA matrix were masked for testing.

Under CV1, 80% of rows in *Y* were used as training set in each round. Under CV2, 80% of columns of *Y* were used as training set. Under CV3, 80% of entries in *Y* were used as training set. We defined new microbes (or diseases) as the microbes (or diseases) without any associated diseases (or microbes). The three cross-validations refer to MDA identification for new microbes, diseases, and microbe–disease pairs, respectively.

We conducted the grid search to find the optimal combination of parameters and found that NLLMDA obtained the best performance when $\gamma_{m}^{\prime }=1$, $\gamma_{d}^{\prime }=1$, γ=0.7, α=0.7, and β=0.1. Sensitivity, specificity, accuracy, and area under the curve (AUC) were applied to evaluate the performance of our proposed NLLMDA method. AUC is the area under Receiver Operating Characteristic (ROC) curve, and the remaining are defined as follows.

(12) $$Sensitivity=\frac{TP}{TP+FN}$$

(13) $$Specificity=\frac{TN}{FP+TN}$$

(14) $$Accuracy=\frac{TP+TN}{TP+FP+TN+FN}$$

where TP, FP, TN, and FN denote true positives, false positives, true negatives, and false negatives, respectively.

### Performance Comparison of Six MDA Prediction Methods

We compared the proposed NLLMDA method with other five MDA identification models, that is, KATZHMDA (Chen et al., 2017), LRLSHMDA (Wang et al., 2017), NGRHMDA (Huang et al., 2017), NTSHMDA (Luo and Long, 2018), and MDLPHMDA (Qu et al., 2019). The five MDA prediction methods separately used the KATZ measurement, Laplacian regularized least squares, neighbor and graph-based recommendation, network topological similarity, and matrix decomposition and label propagation. Tables 1–3 list the performance of these six methods. The best values in each column were denoted in boldface in Tables 1–3. Because we took all unlabeled microbe–disease pairs as negative MDA samples when computing specificity and accuracy, the two measurements are almost the same when accurate to four decimal places on three cross-validations.

**TABLE 1:** Performance comparison of NLLMDA with the other three MDA prediction methods under CV1.

Method	Sensitivity	Specificity	Accuracy	AUC
KATZHMDA	0.2772	0.6690	0.6653	0.3646
LRLSHMDA	**0.3286**	**0.7538**	**0.7496**	**0.4364**
NTSHMDA	0.1899	0.6177	0.6138	0.3042
NGRHMDA	0.0777	0.3423	0.4817	0.4156
MDLPHMDA	0.3273	0.6890	0.6855	0.4022
NLLMDA	0.3218	0.5350	0.5350	0.3120

*The bold values denote the best performance in each column.*

Table 1 shows the sensitivity, specificity, accuracy, and AUC values obtained from KATZHMDA, LRLSHMDA, NGRHMDA, NTSHMDA, MDLPHMDA, and NLLMDA under CV1. From Table 1, we can find that all six MDA prediction methods did not obtain better sensitivity, specificity, accuracy, and AUC under CV1. We thought that it may be resulted in by different structures of data.

Table 2 lists the performance of the six MDA prediction models under CV2. In the cross-validation experiment, NLLMDA computed the best sensitivity and AUC. Especially, NLLMDA outperformed 4.69, 20.42, 9.32, 56.45, and 16.14% compared with KATZHMDA, LRLSHMDA, NTSHMDA, NGRHMDA, and MDLPHMDA, respectively, in terms of sensitivity. NLLMDA outperformed 4.09, 10.46, 8.18, 8.94, and 9.45% compared with the above five methods in terms of AUC. AUC is a more important evaluation metric than the other three metrics. Therefore, NLLMDA obtained better performance and was more appropriate to find associated microbes for a new disease.

**TABLE 2:** Performance comparison of NLLMDA with the other three MDA prediction methods under CV2.

Method	Sensitivity	Specificity	Accuracy	AUC
KATZHMDA	0.8317	0.6487	0.6501	0.8662
LRLSHMDA	0.6944	**0.7333**	0.7330	0.8086
NTSHMDA	0.7913	0.5905	0.5921	0.8292
NGRHMDA	0.3800	0.3285	**0.7403**	0.8224
MDLPHMDA	0.7318	0.6653	0.6658	0.8178
NLLMDA	**0.8726**	0.5592	0.5592	**0.9031**

*The bold values denote the best performance in each column.*

**FIGURE 2:**
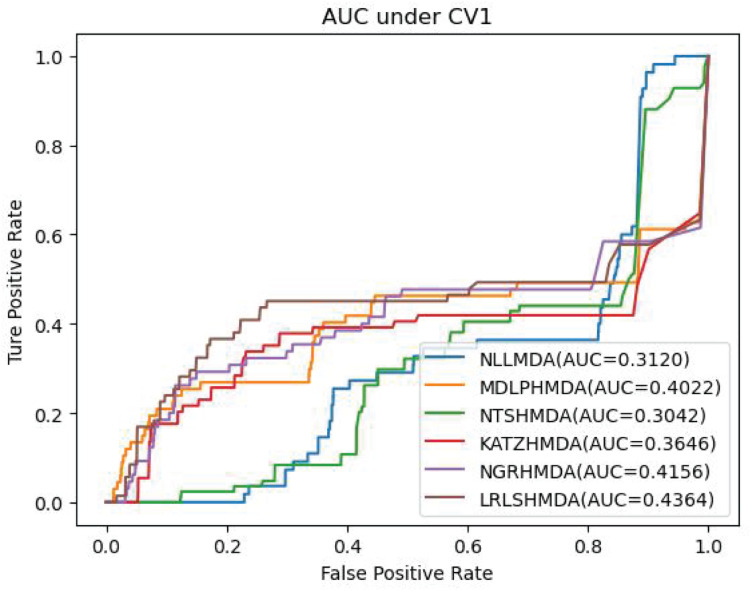
The AUC values of six MDA prediction methods under CV1.

**FIGURE 3:**
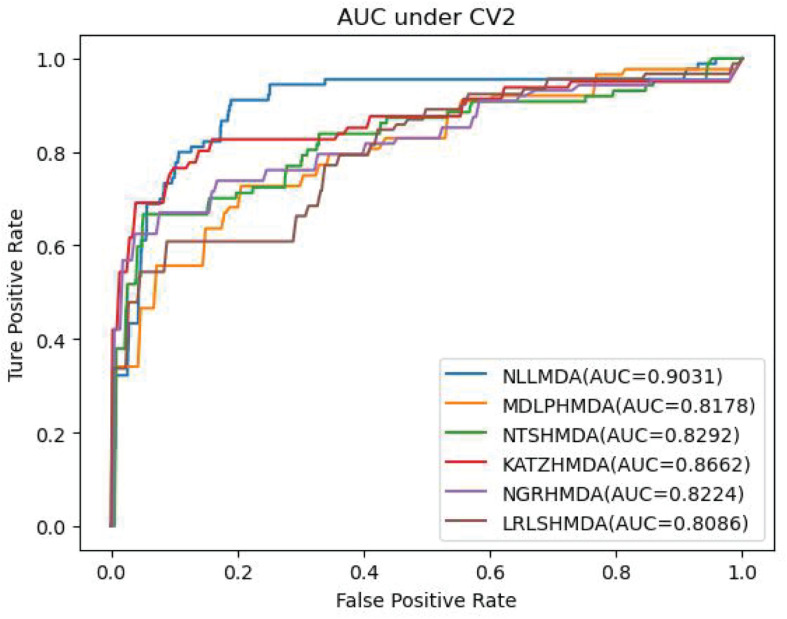
The AUC values of six MDA prediction methods under CV2.

**FIGURE 4:**
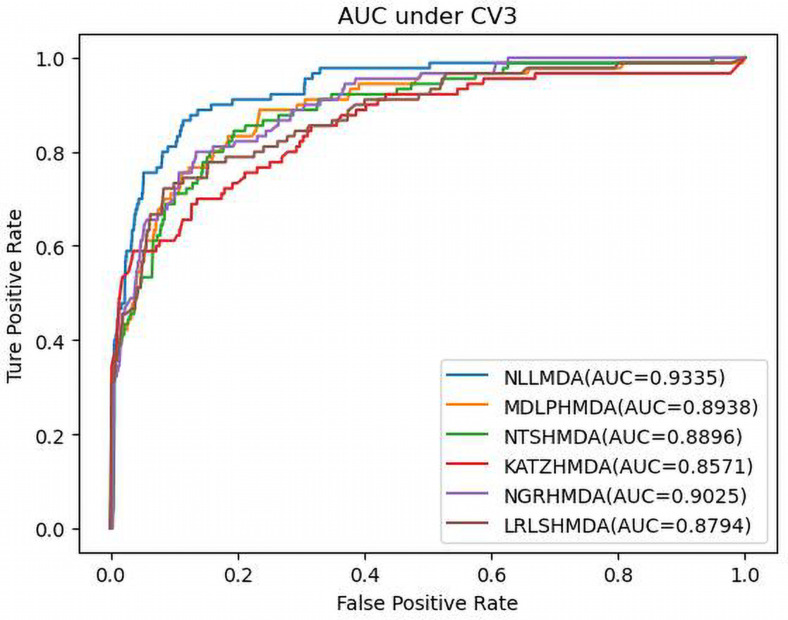
The AUC values of six MDA prediction methods under CV3.

Table 3 shows the predictive results from the proposed NLLMDA method and other five MDA identification methods under CV3. The sensitivity and AUC values of NLLMDA significantly outperformed the other five MDA identification methods. Especially, NLLMDA outperformed 7.84, 11.09, 4.68, 53.07, and 7.77% compared with KATZHMDA, LRLSHMDA, NTSHMDA, NGRHMDA, and MDLPHMDA, respectively, in terms of sensitivity. NLLMDA outperformed 8.18, 5.80, 4.70, 3.32, and 4.25% compared with the above five methods in terms of AUC. AUC is a more important evaluation metric than the other three measurements. Therefore, NLLMDA outperformed the other five MDA prediction models and is an effective MDA prediction method. Figures 2–4 show the AUC values obtained by all six MDA prediction models under three cross-validations.

**TABLE 3:** Performance comparison of NLLMDA with the other three MDA prediction methods under CV3.

Method	Sensitivity	Specificity	Accuracy	AUC
KATZHMDA	0.8262	0.6503	0.6518	0.8571
LRLSHMDA	0.7971	0.7412	0.7416	0.8794
NTSHMDA	0.8545	0.5904	0.5926	0.8896
NGRHMDA	0.4207	0.3308	**0.7796**	0.9025
MDLPHMDA	0.8268	0.6729	0.6741	0.8938
NLLMDA	**0.8965**	0.5600	0.5600	**0.9335**

*The bold values denote the best performance in each column.*

### Case Study

We further analyzed the performance of NLLMDA by two cases. We intend to find the possible microbes associated with colon cancer and colorectal cancer. Although a rare population of undifferentiated cells is closely associated with tumor formation and maintenance, this has not still been found for colon cancer. In addition, colorectal carcinoma has a dense association with specific eating patterns affecting the gut microbiota (Garrett, 2019). The gastrointestinal tract is closely populated with microorganisms. Therefore, we predicted the top 20 microbes associated with the two cancers. The results are shown in Tables 4, 5.

**TABLE 4:** The predicted top 20 microbes associated with colon cancer.

Rank	Microbe	Evidence	DOI
1	Acidobacteriaceae	Unconfirmed	
2	Aeromonadaceae	Confirmed	https://doi.org/10.1002/mnfr.201700554
3	*Anaerovorax*	Unconfirmed	
4	Cellulomonadaceae	Unconfirmed	
5	Thiotrichaceae	Unconfirmed	
6	*Clostridium cocleatum*	Confirmed	https://doi.org/10.1016/S0304-3835(97)04698-3
7	Clostridiaceae	Confirmed	https://doi.org/10.1007/s10620-016-4238-7
8	Peptostreptococcaceae	Confirmed	https://doi.org/10.1007/s10620-016-4238-7
9	Bacillaceae	Unconfirmed	
10	Syntrophobacteraceae	Unconfirmed	
11	Polyangiaceae	Unconfirmed	
12	Desulfovibrionaceae	Confirmed	https://doi.org/10.1080/19490976.2016.1150414
13	*Ruminococcus productus*	Confirmed	https://dx.doi.org/10.3748%2Fwjg.v12.i42.6741
14	Paenibacillaceae	Unconfirmed	
15	Promicromonosporaceae	Unconfirmed	
16	Nitrospiraceae	Unconfirmed	
17	Desulfobacteraceae	Unconfirmed	
18	*Bifidobacterium catenulatum*	Confirmed	https://www.ncbi.nlm.nih.gov/pmc/articles/PMC4171173/
19	Alteromonadaceae	Confirmed	https://doi.org/10.1007/s10620-016-4238-7
20	Prevotellaceae	Unconfirmed	

**TABLE 5:** The predicted top 20 microbes associated with colorectal carcinoma.

Rank	Microbe	Evidence	DOI
1	Proteobacteria	Confirmed	https://doi.org/10.1016/j.ebiom.2019.09.050
2	*Haemophilus*	Confirmed	https://doi.org/10.3892/or.2015.4398
3	*Streptococcus*	Confirmed	https://doi.org/10.1016/j.ebiom.2019.09.050
4	Actinobacteria	Confirmed	https://doi.org/10.1155/2019/8020785
5	*Tannerella*	Unconfirmed	
6	*Eubacterium*	Confirmed	https://doi.org/10.3892/or.2015.4398
7	*Porphyromonas*	Confirmed	https://doi.org/10.1016/j.ebiom.2019.09.050
8	*Lactobacillus*	Confirmed	https://doi.org/10.1080/01635581.2012.700758
9	*Veillonella*	Confirmed	https://doi.org/10.3892/or.2015.4398
10	Betaproteobacteria	Unconfirmed	
11	Bacteroidaceae	Confirmed	https://doi.org/10.1186/s12957-019-1754-x
12	*Faecalibacterium*	Confirmed	https://doi.org/10.1007/s12223-019-00706-2
13	*Eubacterium rectale*	Confirmed	https://doi.org/10.3389/fmicb.2015.00020
14	*Odoribacter*	Confirmed	https://doi.org/10.17235/reed.2015.3830/2015
15	*Phascolarctobacterium*	Unconfirmed	
16	*Roseburia*	Confirmed	https://doi.org/10.1016/j.ebiom.2019.09.050
17	*Eubacterium eligens*	Confirmed	https://doi.org/10.3389/fmicb.2015.00020
18	*Subdoligranulum*	Unconfirmed	
19	Eubacteriaceae	Unconfirmed	
20	*Clostridium*	Confirmed	http://dx.doi.org/10.1590/S1517-838246420140665

Table 4 shows the predicted top 20 microbes associated with colon cancer. The 20 associations are not included in the known MDAs in the HMDAD. There are 8 MDAs validated by recent documents among the 20 MDAs. That is, 40% MDAs have been validated by publications. More importantly, Acidobacteriaceae are able to grow on various sugars or polysaccharides, and some Acidobacteriaceae use amino acids as carbon sources. They grow with a slow speed and grow better under nutrient-limiting conditions. They have been validated to associate with irritable bowel syndrome in the HMDAD (Saulnier et al., 2011). We found that Acidobacteriaceae may associate with colon cancer with the highest linkage probability.

Similarly, Table 5 lists the predicted top 20 microbes associated with colorectal carcinoma. The 20 MDAs are not included in the HMDAD. Among the 20 MDAs, 15 MDAs were reported by related publications. That is, 75% MDAs have been confirmed by documents. In addition, *Tannerella forsythia* is one bacterial pathogen related to human periodontitis, which is a polymicrobial inflammatory disease in tooth-surrounding tissues. It is closely associated with periodontitis, liver cirrhosis, atherosclerosis, and esophageal adenocarcinoma (Jorth et al., 2014; Qin et al., 2014; Bale et al., 2017; Sharma, 2020). The results showed that *Tannerella* may densely link with colorectal carcinoma.

## Discussion

Microbes are commonly distributed in various species and show important role in many biological processes. Many human diseases, for example, intestinal diseases, involved microorganisms. Therefore, finding the potential associations between microbes and diseases can boost the understanding of the pathogenic mechanisms of diseases and its drug research and development.

Traditional experimental methods used for MDA identification are costly and time-consuming. Computational models were designed to uncover new MDAs. However, the prediction performance of computational methods further needs improvement. Therefore, NLLMDA was exploited to find MDA candidates based on negative MDA selection, linear neighborhood similarity, label propagation, information integration, and known biological data. Experimental results showed that NLLMDA obtained better prediction performance. After that, we further analyzed two cases about colon cancer and colorectal carcinoma. We found the top 20 microbes associated with the above two diseases and need to further experimental confirmation.

The proposed NLLMDA methods can obtain better predictive performance. It may be the following characteristics. Firstly, it selected credible negative MDA samples. Secondly, it used linear neighborhood similarity to consider neighborhood information. Thirdly, it conducted information integration based on the prediction results by the computed three similarity scores.

In the future, we will firstly integrate more biological features related to microbes and diseases to more completely reflect the biological information of the two entities. Secondly, we will design more robust algorithms to extract high-quality negative MDA samples. Finally, we will exploit more effective models, such as deep learning, to improve MDA prediction accuracy.

## Data Availability Statement

All datasets generated for this study are included in the article/supplementary material, further inquiries can be directed to the corresponding author/s.

## Author Contributions

JC conceived, designed, and managed the study. YC, CS, and HMS proposed the computational models. YC wrote the manuscript. XS and BJ revised the original draft. HJS and MS discussed the computational models and gave the conclusion. All authors read and approved the final manuscript.

## Conflict of Interest

XS and BJ were employed by the company Genesis Beijing Co., Ltd. The remaining authors declare that the research was conducted in the absence of any commercial or financial relationships that could be construed as a potential conflict of interest.
